# Expression of chromogranin A-derived antifungal peptide CGA-N12 in *Pichia pastoris*

**DOI:** 10.1080/21655979.2020.1736237

**Published:** 2020-03-12

**Authors:** Xiaohua Li, Yong Fan, Qiong Lin, Jianxiong Luo, Yide Huang, Yuwang Bao, Liyu Xu

**Affiliations:** a Department of Respiratory Medicine, Affiliated Fuzhou First Hospital of Fujian Medical University, Fuzhou, Fujian, China; b Central Laboratory, Affiliated Fuzhou First Hospital of Fujian Medical University, Fuzhou, Fujian, China; c Provincial University Key Laboratory of Cellular Stress Response and Metabolic Regulation, College of Life Sciences, Fujian Normal University, Fuzhou, China

**Keywords:** Chromogranin A derived peptide, antimicrobial peptide, *Pichia pastoris*, recombinant expression

## Abstract

The human chromogranin A-derived peptide CGA-N12, which is composed of 12 amino acid residues with the sequence ALQGAKERAHQQ, showed strong antifungal activity and the least hemolytic activity in previous studies. However, synthetic peptides are relatively expensive to produce. Recombinant expression of peptides in the host cells, such as bacteria or yeast, can fastly provide cost-efficient products of peptides. Here, we developed an innovative system to produce CGA-N12 peptides in the yeast *Pichia pastoris* GS115 using genetic engineering technology. In order to directly secret short CGA-N12 peptides into the culture media from GS115 cells and enhance its expression effect, the structure of the CGA-N12 coding sequence was designed to mimic that of native α-factor gene of *Saccharomyces cerevisiae*. Four long primer pairs with sticky end were used to synthesize CGA-N12 expression sequence which contains four copies of CGA-N12 flanked by a Lys-Arg pair and two Glu-Ala repeating units. Endogenous proteases Kex2 and Ste13 in Golgi apparatus recognize and excise Lys-Arg and Glu-Ala pair to release short CGA-N12 peptides from the tandem repeat sequences, respectively. The CGA-N12 peptides were successfully expressed in *Pichia pastoris* with a yield of up to 30 mg/L of yeast culture as determined using HPLC. Our study indicated that the strategy employed in this work may be a good way to express small-molecule peptides directly in *the Pichia pastoris* system.

## Graphical Abstract

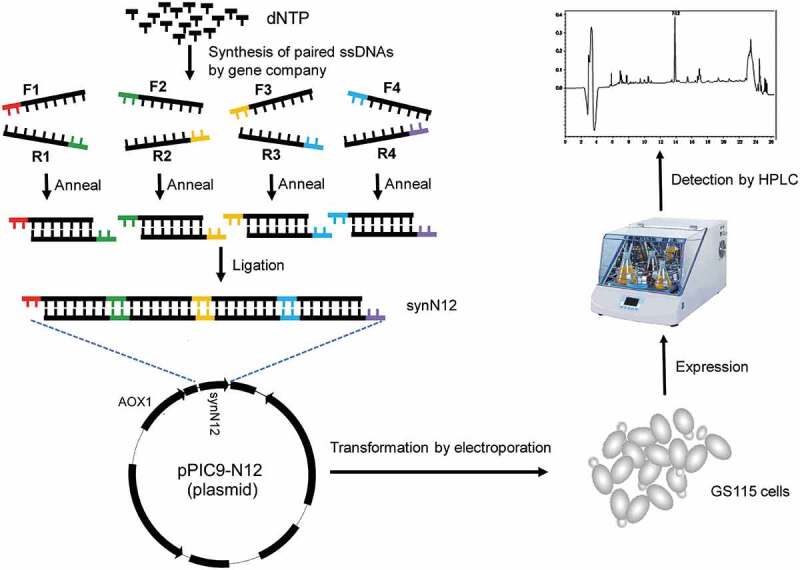

## 1. Introduction

In nature, response to the challenge of pathogens is essential for the survival of all living organisms including human. Over the past decades, the overuse of antibiotics, such as β-lactams, aminoglycosides, streptogramins, chloramphenicols etc., has led to the emergence of antibiotic-resistant microorganism [1,2]. The need for finding novel and effective alternatives is urgent. Antimicrobial peptides (AMPs) are part of the inherent immune system. Many animals and plants can naturally produce AMPs which act as one immediate protective barrier against infection of pathogens [3,4]. Most AMPs are cationic and hydrophobic molecules with direct antimicrobial activity via membrane target, DNA target, RNA target or protein target [5–9]. These AMPs with different antimicrobial mechanisms to antibiotics are attracting our increasing attention to develop novel antimicrobial drugs for defending antibiotic-resistant microorganism.

The human chromogranin A (CGA) is a protein consisting of 436 amino acids with a molecular weight of approximately 42–53 kDa, and stored and released from most endocrine and neuroendocrine cells and neurons. CGA is the precursor of several functional peptides with different biological activities [10,11]. The natural N-terminal 1–76 CGA-derived fragment in bovine sequence, named vasostatin-1, was found to display antimicrobial activity against Gram-positive bacteria, filamentous fungi and yeast cells at micromolar concentrations [12]. The sequence Arg47-Leu66 at the C-terminal region of vasostatin-I is the most active shortest peptide [13]. Li et al. studies elucidated that human CGA-N46 peptide, harboring in the N-terminus of human CGA protein from the 31st to 76th amino acid, had anti-Candidal activity and the highest antagonistic activity to *C. krusei* [14]. They tried to explore the mechanism of CGN-N46 against *C. krusei*, and found that the CGN-N46 peptide could inactivate the *Taq* DNA polymerase *in vitro* and decreased the fluorescence of dichlorofluorescein (DCF) (used for detecting reactive oxygen species) and probe Rh-123 fluorescence (used for detecting mitochondria membrane potential) in a concentration-dependent manner, indicating that the anti-Candidal mechanism of CGA-N46 could be that the CGA-N46 peptide inhibited the growth of Candida species by inhibiting DNA synthesis and decreasing intracellular reactive oxygen species (ROS) levels and mitochondria membrane potentials [15,16]. The derivatives of CGA-N46 (CGA-N16, CGA-N15, CGA-N12 and CGA-N8) designed by successively deleting amino acids of CGA-N46 showed higher antifungal activities than CGA-N46 itself. Based on the predicted physicochemical properties of the peptides, the instability index of CGA-N16, CGA-N15, CGA-N12 and CGA-N8 was 91.88, 55.03, 38.23 and −12.48, respectively, which indicated that the CGA-N12 and CGA-N8 were more stable than CGA-N15 and CGA-N16 [17]. The peptide CGA-N12 also showed the least hemolytic activity [17].

Due to its high potential as a new anti-Candidal peptide, a large amount of CGA-N12 peptides are required for the aim of researches or clinical applications. However, synthetic peptides are relatively expensive to produce. Recombinant expression in the host cells, such as bacteria or yeast, can fastly provide cost-efficient products of peptides. The aim of this study was to develop an efficient method for expressing CGA-N12 peptide using *Pichia pastoris* host. To achieve this, we imitated expression construction of the native α-factor, the sexual hormone of *Saccharomyces* cerevisiae, to design expression sequence with four copies of CGA-N12 in *Pichia pastoris*. We expected that the expressed polypeptide could be processed by endogenous proteases Kex2 and Ste13 to release the mature CGA-N12 peptides in the fermentation supernatant.

## 2. Materials and methods

### 2.1. Strains

*Escherichia coli* Top10′ was used as a cloning host. *Pichia pastoris* GS115 (*his^−^*, mut^+^) was used as a host for expression of recombinant N12 peptide. Luria-Bertani (LB) medium (1% tryptone, 1% NaCl and 0.5% yeast extract) was used for the growth of Bacteria Top10′.

#### 2.1.1. Design and construction of expression vector

To express the CGA-N12 peptide in *Pichia pastoris*, a codon-optimized sequence with four tandemly arranged copies flanking a Kex2 and two Ste13 sites was designed. For synthesizing codon-optimized sequence, three pairs of long oligos with about 75 nt were designed (Table 1) and synthesized by Sangon Biotech company (Sangon Biotech, Shanghai, China). The oligos are flanked by 4 nt sticky ends for facilitating ligation. The oligos were resuspend in sterile deionized water to a concentration of 20 μM. Forward and reverse oligos for each pair were mixed in a tube and incubated for 4 min at 95°C in a beaker of boiling water. Then, the beaker was removed from the flame and allowed the water to cool to room temperature to anneal the oligos. Three annealed oligos were mixed with equal volume and ligated at 16°C overnight by T4 DNA ligase (TAKARA Bio, Beijing, China) to form the full coding sequence of CGA-N12. To clone the full coding sequence into pPIC9, firstly, the pPIC9 was digested with *Xho* I and *Eco*R I enzymes, and then digested pPIC9 was purified with gel extraction kit (Promega, Shanghai, China). Full coding sequence of CGA-N12 was cloned into digested pPIC9 by T4 DNA ligase in a condition according to user’s manual. Top 10′ cells were used to transform the expression vector (named as pPIC9-N12). Finally, expression vector construct was verified by DNA sequencing.

**Table 1.** Sequences of long oligos for synthesizing full coding sequence of CGA-N12.

Oligo name	Sequences (5′-3′)
Primer pair 1 forward	TCGAGAAACGAGAGGCTGAAGCTGCTCTTCAAGGTGCCAAAGAACGAGCTCACCAGCAGAAACGAGAGGCTGAAG
Primer pair 1 reverse	GCAGCTTCAGCCTCTCGTTTCTGCTGGTGAGCTCGTTCTTTGGCACCTTGAAGAGCAGCTTCAGCCTCTCGTTTC
Primer pair 2 forward	CTGCTCTTCAAGGTGCCAAAGAACGAGCTCACCAGCAGAAACGAGAGGCTGAAGCTGCTCTTCAAGGTGCCAAAG
Primer pair 2 reverse	CGTTCTTTGGCACCTTGAAGAGCAGCTTCAGCCTCTCGTTTCTGCTGGTGAGCTCGTTCTTTGGCACCTTGAAGA
Primer pair 3 forward	AACGAGCTCACCAGCAGAAACGAGAGGCTGAAGCTGCTCTTCAAGGTGCCAAAGAACGAGCTCACCAGCAGTAAG
Primer pair 3 reverse	AATTCTTACTGCTGGTGAGCTCGTTCTTTGGCACCTTGAAGAGCAGCTTCAGCCTCTCGTTTCTGCTGGTGAGCT

### 2.2. Generation of Pichia pastoris transformants

*Pichia pastoris* GS115 was used as a host to expression recombinant CGA-N12 peptide. For the preparation of competent cells, GS115 was grown in 5 mL of YPD medium in a 50 mL conical at 30°C overnight, and then inoculated 500 mL of fresh medium in a 2 L flask with 0.5 mL of the overnight culture. The fresh medium with GS115 was allowed to grow overnight again to an OD_600_ = 1.3–1.5. The cell pellet of GS115 was harvested by centrifuging the cultured cells at 1500 g for 5 min at 4°C and resuspended with ice-cold sterile water. After removing the water by centrifugation, the pellet was resuspended in 20 mL of ice-cold 1 mol/L sorbitol, and then the cells were centrifuged and resuspended in 1 mL of ice-cold 1 mol/L sorbitol to get competent cells. For electroporation of GS115, the 80 μL of cells treated with sorbitol were mixed with 10 μg of linearized recombinant plasmid pPIC9-N12 (linearized by *Sac* I) and transfer them to an ice-cold 0.2 cm electroporation cuvette. Before electroporation in Bio-Rad Gene Pulser Xcell (Bio-Rad USA), the cells were incubated in the cuvette on ice for 5 min. The parameter of electroporation is 1500 V, 25 μF, 200 Ω. After the cells were pulsed, 1 mL of ice-cold sorbitol was immediately added into the cuvette, and then the cells were spread on MD plates (2% dextrose, 1.34% yeast nitrogen base, 400 μg/L biotin and 2% agar) to screen transformants.

### 2.3. PCR analysis of integrants

To further confirm the integration of CGA-N12 gene into the genome of GS115 growing on MD plates, genomic PCR assay was employed. Briefly, the clones growing on MD plates were cultured in the YPD medium and their genomic DNA was isolated using Rapid Yeast Genomic DNA Isolation Kit (Sangon Biotech, Shanghai, China). Three hundred nanograms of genomic DNA was used as a template to verify the integration of N12 gene by PCR using the 5′ *AOX1* primer (5′-GACTGGTTCCAATTGACAAGC-3′) paired with the 3′ *AOX1* primer (5′-GCAAATGGCATTCTGACATCC-3′). For amplification controls, 10 ng of recombinant plasmid pPIC9 K-N12 and 300 ng of genomic DNA of GS115 were used as positive control and negative control, respectively.

### 2.4. Expression of recombinant CGA-N12 peptide in Pichia pastoris

Three groups of media are used for expressing secreted proteins in *Pichia pastoris*. They are BMGY/BMMY (buffered complex glycerol or methanol medium), BMG/BMM (buffered minimal glycerol or methanol medium) and MGY/MM (minimal glycerol or minimal methanol medium). BMGY/BMMY is usually used for the expression because the inclusion of yeast extract and peptone in the medium act as a ‘mixed feed’ allowing better growth and biomass accumulation. In this study, two groups of media, BMGY/BMMY and BMG/BMM were employed to express CGA-N12 peptide. The GS115 integrants were culture in BMGY (1% yeast extract, 2% peptone, 1% glycol, 400 μg/L biotin, and 0.1 M potassium phosphate pH 6.0) or BMG (1.34% yeast nitrogen bases, 1% glycol, 400 μg/L biotin, and 0.1 M potassium phosphate pH 6.0) for the growth at 30°C in a shaking incubator (250 rpm) until culture reached an OD_600_ = 3.0–5.0, and then harvested by centrifugation. The supernatant was decanted and the cells resuspend to an OD_600_ of 1.0 in BMMY (1% yeast extract, 2% peptone, 400 μg/L biotin, 1% methanol, and 0.1 M potassium phosphate pH 6.0) or BMM (1.34% yeast nitrogen bases, 1% methanol, 400 μg/L biotin, and 0.1 M potassium phosphate pH 6.0) medium to induce expression. One hundred percent methanol was added to a final concentration of 1% methanol every 24 h to maintain induction for 96 h. After induction, the supernatant was separate from the cell pellets by centrifugation at 3000 g for 5 min at 4°C and was freeze-dried for further experiment.

### 2.5. HPLC analysis

An HPLC system (Agilent Technologies, Waldbronn, Germany) equipped with a quaternary pump, an automatic injector, and a UV detector was used to detect the expression of CGA-N12 in fermentation supernatant. A column SHIMADZU Inertsil ODS-SP (4.6 × 250 mm × 5 μm) from Shimadzu (Guangzhou, China) was employed. Mobile phases were 0.1% trifluoroacetic (TFA) in water (v/v) (pump A) and 0.1% TFA in 100% acetonitrile (ACN) (v/v) (pump B). The elution was performed with a linear ACN gradient. Elution gradient was 0–30% B for 20 min, 95% B for 10 min at a flow rate of 1 mL/min, and the separation temperature was set at 25°C. The injected volume was 60 μL, and detection wavelengths were set at 214 nm.

## 3. Results

### 3.1. Design of a four copy coding sequence of CGA-N12

**Figure 1.**
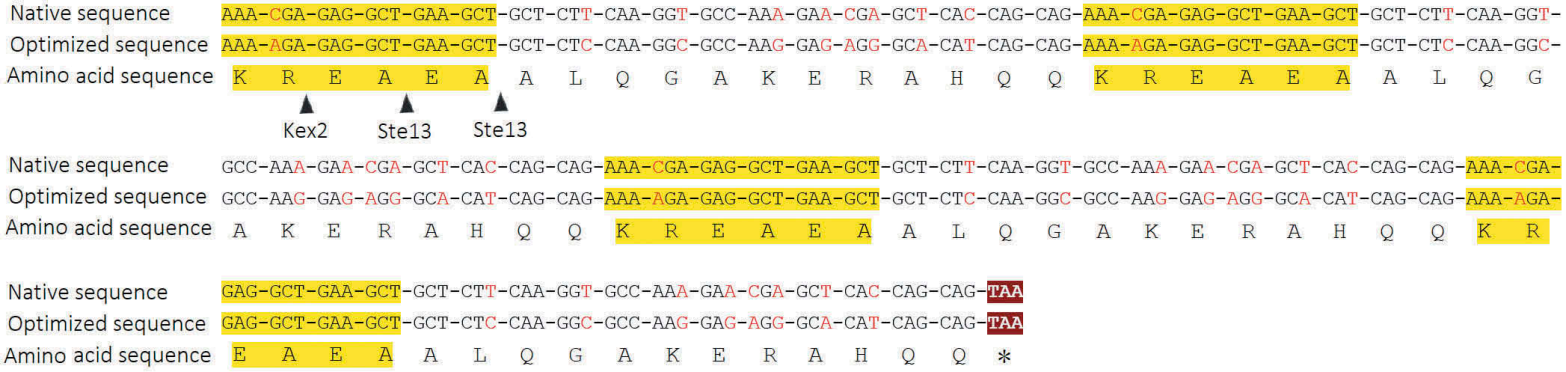
Design of coding sequence of chromogranin A-derived peptide CGA-N12 with four copies. Yellow highlight color showed processing sites of Kex2 and Ste13 proteases. Red letters showed the difference of codon between native sequence and optimized sequence.

Many peptide hormones and neuropeptides are synthesized as precursors, which undergo proteolytic processing to release the mature peptides through the secretory pathway of endoplasmic reticulum (ER) and Golgi apparatus. The α-factor, a 13-amino-acid mating pheromone, is also synthesized as part of a larger precursor by yeast of the α mating type. The α-factor precursor consists of a pre-peptide, a pro-peptide and four copies of mature α-factor mating pheromone [18]. The pre-peptide is used as a signal peptide to guide the precursor to ER lumen. The pro-peptide facilitates the transport of precursors from ER to Golgi apparatus. The four mature α-factor peptides are separated by a processing site consisting of a Lys-Arg pair and a spacer sequence of several Glu-Ala repeating units [18]. Kex2 protease locating in Golgi apparatus first cleaves the precursor on the carboxyl side of the Arg residues of the Lys-Arg pair, and Ste13, a dipeptidyl aminopeptidase, then excise the Glu-Ala repeating units to generate the mature α-factor peptide. Based on the expression of α-factor mating pheromone in the yeast, we planned to mimic the natural strategy of α-factor production to express CGA-N12 in *Pichia pastoris*. The four CGA-N12 peptide sequences were flanked by a Lys-Arg pair and two Glu-Ala repeating units. In order to better express CGA-N12, the codons were optimized according to the codon usage bias of *Pichia pastoris*. Finally, a total of 32 non-preferred codons in the four copies of the CGA-N12 coding sequence were replaced by the *Pichia pastoris* preferred codons (Figure 1).

### 3.2. Synthesis of coding sequence of synN12 and construction of recombinant plasmid

**Figure 2.**
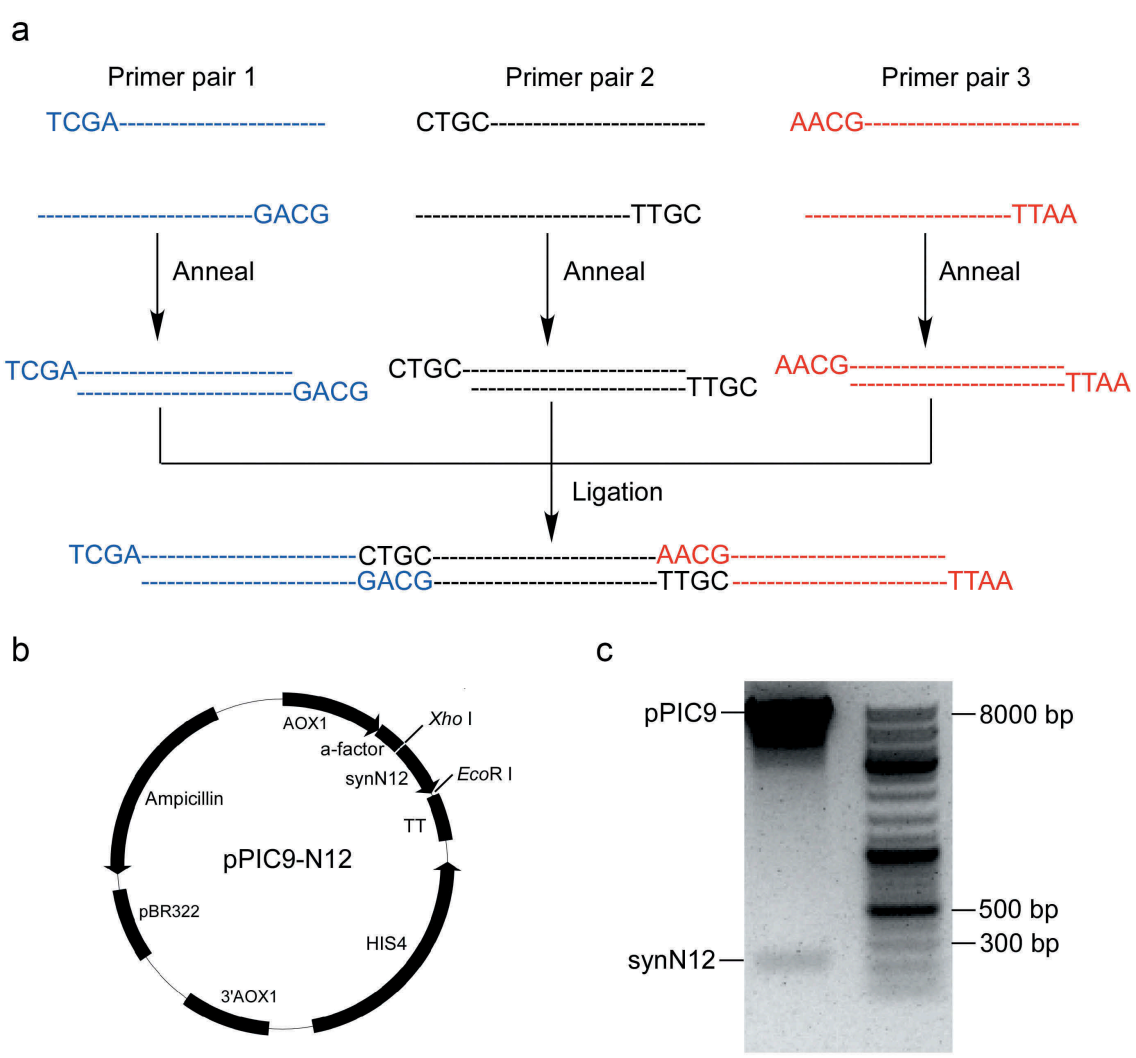
Synthesis of synN12 and construction of expression vector pPIC9-N12. a) Schematic diagram of synthesizing full coding sequence of CGA-N12. b) Construct of expression vector pPIC9-N12. c) Identification of expression vector pPIC9-N12 digesting by restriction enzymes.

The coding sequence expressing four copies of CGA-N12 peptides is only 219 bp with stop codon. To synthesize the optimized coding sequence of CGA-N12 peptides (here named with synN12), three long primer pairs were designed. The plus and minus primers were annealed to form three short double-strand DNA fragments, and the three fragments were finally ligated into an integrated expressing sequence. Figure 2(a) is the schematic diagram of synthesizing the sequence of synN12. The synN12 sequence was cloned into the expression plasmid pPIC9, resulting in a construct named pPIC9-N12 (Figure 2(b)). Recombinant plasmid was verified by digestion using restriction enzymes (Figure 2(c)) and finally confirmed by DNA sequencing.

### 3.3. Expression of CGA-N12 in Pichia pastoris

**Figure 3.**
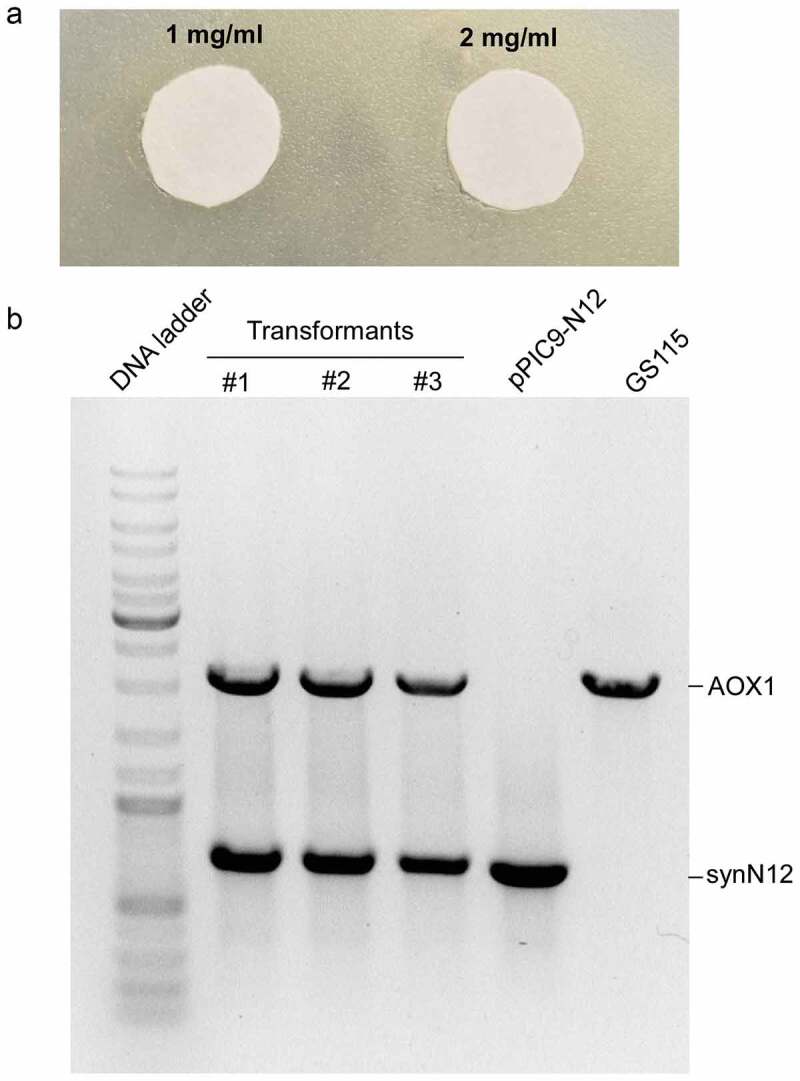
The toxicity of CGA-N12 toward *Pichia pastoris* and analysis of transformants by genomic PCR. a) The inhibition zone test to check the toxicity of CGA-N12 toward *Pichia pastoris*. b) PCR analysis of transformants. Plasmid pPIC9-N12 (lane 5) or GS115 genomic DNA (lane 6) as template were employed as controls. #1, #2, and #3 represented three different transformants using in this study.

CGA-N12 peptide is an antifungal peptide and can inhibit the growth of fungi *Candida* species. The yeast *Pichia pastoris* also belongs to fungus. CGA-N12 peptide may also inhibit the growth of *Pichia pastoris*. Therefore, before CGA-N12 was recombinantly expressed in *Pichia pastoris*, we checked the toxicity of CGA-N12 toward *Pichia pastoris* using synthetic CGA-N12 peptide (Sangon Biotech, Shanghai, China). The inhibition zone test was performed and 1 mg/mL and 2 mg/mL of CGA-12 peptide were used to check the toxicity of the peptide toward *Pichia pastoris*. The result showed that CGA-N12 did not show the toxicity toward *Pichia pastoris* GS115 even at 2 mg/mL concentration (Figure 3(a)), indicating that *Pichia pastoris* GS115 is suitable as a host to express recombinant CGA-N12 peptide. After linearized pPIC9-N12 plasmids were transformed into GS115 by electroporation, transformants were firstly screened in MD plates. Several colonies on the MD plates were picked to culture in BMGY medium and their genomic DNAs were isolated. PCR was employed to further analyze the integration of synN12 into the GS115 genome using AOX1 primers. Both *AOX1* and target genes would be amplified if the target gene was successfully integrated into the GS115 genome (Figure 3(b)).

**Figure 4.**
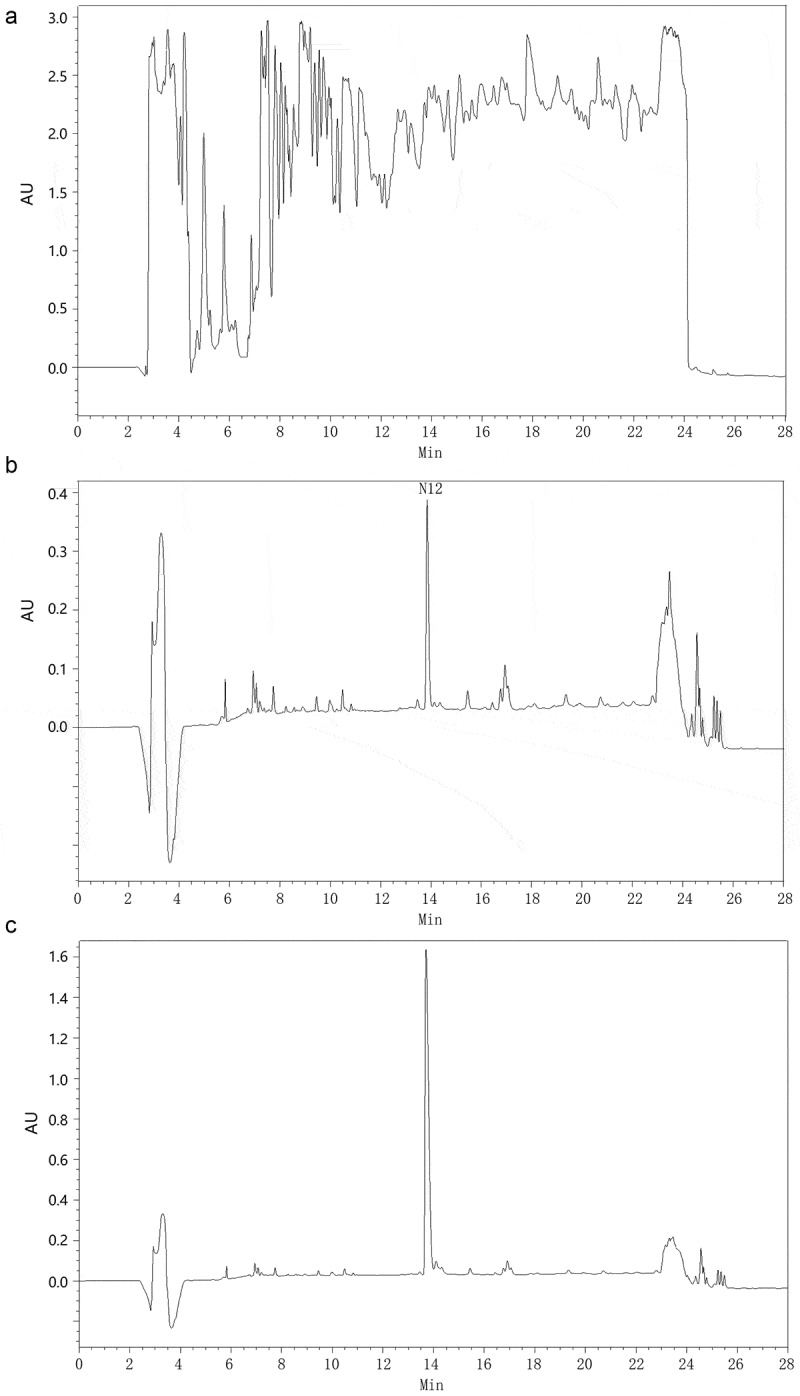
Expression of recombinant CGA-N12 in Pichia pastoris. a) HPLC analysis of fermentation supernatant culturing in BMMY medium. b) HPLC analysis of fermentation supernatant culturing in BMM medium. c) HPLC analysis of synthetic CGA-N12 peptide used as the internal standard. Synthetic CGA-N12 peptide was added into BMM medium to reach the concentration of 1 mg/mL, and 60 μL of the sample was loaded into HPLC column for separation.

BMMY and BMM were used as fermentation media to express recombinant CGA-N12 peptide in this work. The signal of recombinant CGA-N12 expressed in the BMMY medium was masked by these signals of peptides existed in medium (Figure 4(a)). A significant signal peak at a retention time of 14 min in chromatographic column was observed in the BMM medium (Figure 4(b)). The peak at a retention time of 14 min was identified as the signal of CGA-N12 using synthetic CGA-N12 peptide as the internal standard (Figure 4(c)). As a result, the expression level of recombinant CGA-N12 in the BMM medium reached about 30 mg/L.

## 4. Discussion

Pathogenic microorganisms with Antibiotic resistance are one of the most challenging global health threats in our society. This situation has driven us to search for novel types of antimicrobial agents. AMPs represent promising alternatives to conventional antibiotics for the treatment of drug-resistant infections. However, their applications are limited by their high manufacturing cost. The production of AMPs in a cost-effective and scalable method is challenging. Engineering living organisms using recombinant DNA techniques represent a promising approach to produce AMPs in an inexpensive manner. Numerous AMPs have been successfully produced in various host cells, such as *Escherichia coli* [19], *Lactococcus lactis* [20], the plant tobacco [21], *Pichia pastoris* [22]. Among these host cells, *Pichia pastoris* has become one of the most favorable recombinant expression systems because it has both prokaryotic and eukaryotic characteristics. Similar to the bacterium *Escherichia coli, Pichia pastoris* grows rapidly and is amenable to high-cell-density fermentations [23]. Like mammalian cells, the expressed products in *the Pichia pastoris* system can be modified after translation into the secret pathway, by processes disulfide bond formation [24], proteolytic cleavage [25] and glycosylation [26].

Small-molecule AMPs were often expressed as fusion proteins [22,27]. A disadvantage of fusion protein expression for AMPs is that fusion proteins need to be processed *in vitro*, by proteases or chemicals, to release AMPs. In this work, we tried to develop an innovative approach to directly express small-molecule AMP instead of fusion proteins in *Pichia pastoris* system. Many fungi can secret small-molecule peptides into the environment for communication as pheromone. For example, the α mating type cells of *Saccharomyces cerevisiae* can efficiently secret a sex-specific oligopeptide pheromone called α-factor. Mature α-factor is a small-molecule polypeptide with 13 amino acid residues [28]. The number of amino acids of CGA-N12, a 12-amino-acid peptide, is similar to that of α-factor mature peptide. α-factor was not only found in the yeast *Saccharomyces cerevisiae* but also in other fungi including the yeast *Pichia pastoris* [29]. The organization of all α-factor genes is similar. They are synthesized as part of larger precursors. The α-factor precursors consist of pre-peptide, pro-peptide and several copy mature α-factor peptides. Each α-factor peptide is flanked by processing sites of Kex2 and Ste13 proteases which is located in Golgi apparatus. In order to express small-molecule CGA-N12 peptide successfully in *Pichia pastoris*, the structure of the expression sequence of CGA-N12 was designed to mimic the structure of native α-factor gene of *Saccharomyces cerevisiae*. Our results showed that CGA-N12 peptide was successfully expressed in *Pichia pastoris*. The strategy employed in this study could be a good way to express small-molecule peptides in *Pichia pastoris*.

In *Pichia pastoris* system, the optimization of codon usage and fermentation conditions are the effective strategies to improve the yield of products [26]. To increase the expression level, the CGA-N12 code was optimized according to the codon preference of *Pichia pastoris*. BMMY medium is usually used to express recombinant proteins because *Pichia pastoris* cells grow faster in BMMY medium. We could not detect the CGA-N12 signal by HPLC when the BMMY medium was employed to express the CGA-N12 peptide. The components in the BMMY medium were highly absorbent in UV, which totally mask the signal of CGA-N12 peptide. Another disadvantage expressing small-molecule peptides in BMMY medium was that the peptide components belonging to yeast extract and peptone could interfere with the purification of target peptides. The CGA-N12 signal could easily be detected in the BMM medium, a chemical-defined medium, by HPLC. The chemical defined medium like BMM might be a good choice to express small-molecule peptides in *Pichia pastoris*.

## 5. Conclusions

In conclusion, this study showed that the human chromogranin A-derived peptide CGA-N12 was successfully expressed with a yield of up to 30 mg/L of yeast culture in *Pichia pastoris* GS115. Designing the coding sequences to mimic that of the native α-factor gene of *Saccharomyces cerevisiae* might be a good choice to directly express small-molecule AMPs from *Pichia pastoris* cells.

## Highlights

- Human chromogranin A-derived peptide CGA-N12 was successfully expressed in the yeast *Pichia pastoris.*
- The yields of CGA-N12 were up to 30 mg/L of yeast culture in *Pichia pastoris*.
- BMM medium was more favorable than BMMY medium for detecting the expression of small-molecule CGA-N12 using HPLC assay.
